# Morphology of the unique egg cases of hornsharks (Heterodontiformes: Heterodontidae)

**DOI:** 10.1111/jfb.15945

**Published:** 2024-10-15

**Authors:** Helen L. O'Neill, Kotaro Tokunaga, William T. White

**Affiliations:** ^1^ CSIRO National Research Collections Australia‐Australian National Fish Collection Hobart Tasmania Australia; ^2^ Ibaraki Prefectural Oarai Aquarium Ibaraki Japan

**Keywords:** comparative morphology, egg cases, *Heterodontus*, hornshark, oviparous, taxonomy

## Abstract

Many of the egg cases of oviparous chondrichthyans remain unknown and undescribed in the literature. Egg cases can be a useful taxonomic character for species distinction and can be a valuable indicator of a species distribution in the field. In this study, the egg cases for 9 of the 10 nominal species of *Heterodontus* are described and compared, and the terminology and methodology for studying them are standardized. *Heterodontus* egg cases are distinct and easily identifiable from other oviparous egg cases by having a unique corkscrew shape formed by a pair of lateral keels spiraling along its length. *Heterodontus* egg cases range between 7.5 and 14.5 cm in egg case length, 3.7 and 5.8 cm in egg case width at midportion, and have 0.75–4 complete rotations. Morphometric measurements of egg cases from the nine species were subjected to multivariate analysis, with unique characters enabling distinction between them. Egg cases can be separated into three morphotypes: the “wide keels lacking tendrils” group, the “narrow keels with tendrils” group, and the “wide keels with tendrils” group. The egg case of *Heterodontus ramalheira* remains unknown.

## 1 INTRODUCTION

Egg cases of oviparous chondrichthyans are diverse in their shape and form. Their uniqueness provides valuable taxonomic characters for species distinction and identification (Bustamante et al., 2013; Ishihara et al., 2012; O'Neill et al., 2020; Porcu et al., 2017; Treloar et al., 2006). Generating a reference collection of accurately identified egg cases provides an important foundation for improved understanding of oviparous chondrichthyans. Accurately identified egg cases containing embryos can provide important biological information, such as reproductive cycles and seasonality, variations in fecundity (Palm et al., 2011), embryonic development rates (Hoff, 2009), incubation times (Benjamins et al., 2021), improved knowledge on species distributions (e.g., the Great Eggcase Hunt) (Ellis et al., 2024), and the discovery of essential fish habitat nursery areas (Amsler et al., 2015; Dodd et al., 2022; Hoff, 2016; Maguire et al., 2023). For example, the collection of unassignable egg cases in 1983 laid the foundation for the discovery of a new species, the ridged‐egg catshark *Apristurus ovicorrugatus* White, O'Neill, Devloo‐Delva, Nakaya & Iglésias, 2023, 40 years later (Human, 2011; White et al., 2023). Similarly, three empty egg cases collected in the Antarctic seas were used to describe the dark‐mouth skate *Bathyraja arctowskii* (Dollo, 1904), but it wasn't until almost a century later that a near‐term embryo contained within an egg case became available, providing the crucial connection between the species name based on the initial empty egg cases and the actual skate itself (Stehmann et al., 2021). The identification of egg cases remains challenging as (1) they are often collected independently from the more readily identifiable female that deposited them, (2) there is a lack of morphological descriptions for most chondrichthyan egg cases, and (3) many species are poorly represented in biological collections as they occur in remote, under sampled areas, particularly in deep waters.

The egg cases of oviparous chondrichthyans come in a variety of morphotypes, one of the most unique are the corkscrew‐like egg cases of heterodontid sharks. There are currently 10 recognized nominal species of *Heterodontus*: hornshark *Heterodontus francisci* (Girard, 1855) from the eastern Pacific; crested bullhead shark *Heterodontus galeatus* (Günther, 1870) from eastern Australia; Japanese bullhead shark *Heterodontus japonicus* de Miklouho Maclay & Macleay, 1884 from the northwest Pacific; painted hornshark *Heterodontus marshallae* White et al., 2023 from the eastern Indian Ocean off northwestern Australia; Mexican hornshark *Heterodontus mexicanus* Taylor & Castro‐Aguirre, 1972 from the eastern Pacific; Oman bullhead shark *Heterodontus omanensis* Baldwin, 2005, from the northwestern Indian Ocean; Port Jackson shark *Heterodontus portusjacksoni* (Meyer, 1793) from southern Australia; Galapagos bullhead shark *Heterodontus quoyi* (Fréminville, 1840) from the eastern Pacific; whitespotted bullhead shark *Heterodontus ramalheira* (Smith, 1949) from the western Indian Ocean; and zebra bullhead shark *Heterodontus zebra* (Gray, 1831) from the western Pacific. Despite the egg cases of heterodontid sharks being easily distinguishable from those of other genera due to their corkscrew shape, detailed descriptions and comparisons within the genus have not been made. In this study, egg cases of 9 of the 10 heterodontid species sourced from collections and aquariums were examined to provide detailed description and comparison.

## 2 MATERIALS AND METHODS

### 2.1 Ethical statement

Egg case specimens came from various sources, including those laid by captive females at Ibaraki Prefectural Oarai Aquarium, Japan, and Seahorse World, Beauty Point, Australia. No specimens were euthanized during this study. All egg cases obtained from aquaria were non‐viable. All other egg cases were examined from the holdings of museum collections from the previously collected samples.

### 2.2 Materials examined

Museum abbreviations follow Fricke & Eschmeyer (2024). All specimens are wet except for *H. portusjacksoni* BMNH 1920.10.11.1, which is dried.

*Heterodontus francisci* egg cases (*n* = 7): CSIRO H 9198‐02 (*n* = 2), laid by captive female at Birch Aquarium, San Diego, California; CSIRO H 9198‐01 (*n* = 3), laid by captive female(s) at Birch Aquarium, San Diego, California; unregistered (*n* = 2), laid by captive female(s) from Ibaraki Prefectural Oarai Aquarium, Japan.

*Heterodontus galeatus* egg cases (*n* = 5): AMS IB.7623, Malaney's Beach, Batemans Bay, New South Wales, 35° 44′ S, 150° 15′ E; AMS I.12925, Eden, New South Wales, 37° 04′ S, 149° 55′ E, Australia, collected 1913; AMS IA.5465, La Perouse, Sydney, New South Wales, 33° 59′ S, 151° 12′ E; AMS I.4781, Coogee Bay, New South Wales, 33° 56′ S, 151° 16′ E; AMS IA.8054, Moa Island, Queensland, Australia, 10° 11′ S, 142° 16′ E.

*Heterodontus japonicus* egg cases (*n* = 2): unregistered (*n* = 2), laid by captive females from Ibaraki Prefectural Oarai Aquarium, Japan.

*Heterodontus marshallae* egg case (*n* = 1): NTM S.18275‐001 (paratype), southeast of Evans Shoal, Arafura Sea, Northern Territory, Australia, 10° 08′ S, 130° 05′ E, 131 m depth, collected October 1, 1998.

*Heterodontus mexicanus* egg cases (*n* = 4): CAS‐ICH 040578, northeast Pacific, Baja California, 25° 06′ N, 110° 49′ W, Mexico; CAS‐ICH 232102, Gulf of Mexico, collected May 26, 1974; SIO 65–257 (*n* = 2), Nahia de La Paz, Baja California Sur, Mexico, 24° 19′ N, 110° 27′ E, 79 m depth, collected July 5–6, 1965.

*Heterodontus omanensis* egg case (*n* = 1): removed from female holotype UW 47592, Arabian Sea, Central Oman, Gulf of Masira, northeast of Ra's al Madrakah, collected July 29, 1989.

*Heterodontus portusjacksoni* egg cases (*n* = 6): CSIRO H 8732‐01, CSIRO H 8732‐02, and CSIRO H 8732‐03, laid by captive females at Seahorse World, Beauty Point, Tasmania, Australia, January 2009; CSIRO H 7010‐01, Huskisson Beach, New South Wales, Australia, 35° 02′ S, 150° 40′ E, collected October 10, 2009; AMS I.1417, Bondi, New South Wales, Australia, 33° 54′ S, 151° 17′ E; BMNH 1920.10.11.1 (dry specimen, not measured) near Broome, Western Australia.

Neonate (*n* = 1): NTM S.00043‐001, female 234 mm total length (TL), York Sound Western Australia, collected June 1975.

*Heterodontus quoyi* egg case (*n* = 1): USNM 127779, Lobos de Tierra Bay, ~6° 16′ S, 80° 31′ W, Peru.

*Heterodontus zebra* egg case (*n* = 3): KAUM I.69456, off Oton, Panay Island, Philippines, 10° 37′ N, 122° 14′ E, collected March 1, 2005; unregistered (*n* = 2), laid by captive female(s) from Ibaraki Prefectural Oarai Aquarium, Japan.

### 2.3 Morphological measurements

**FIGURE 1:**
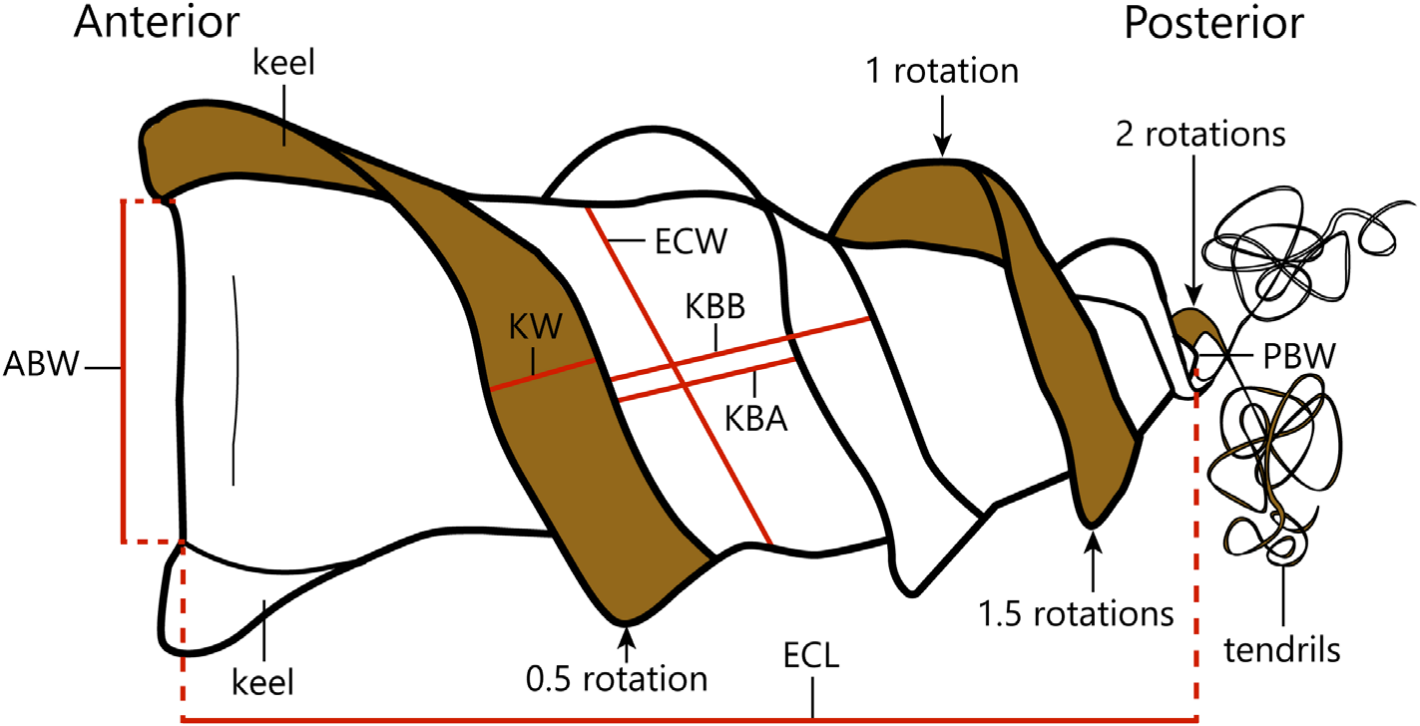
Illustration of the features and measurements of a generic heterodontid egg case in dorso‐ventral view. KW, KBB, and KBA measurements are taken on opposing dorso‐ventral surfaces (each having two measurements). Brown keel highlights the corkscrew nature of the egg case. Number of rotations calculated by following the upper keel and counting the number of turns, see arrows as an example. ABW, anterior border width; ECL, egg case length; ECW, egg case width at midportion; KBA, keel base to adjacent keel apex at midportion; KBB, keel base to adjacent keel base at midportion; KW, keel width at midportion; PBW, posterior border width.

When in utero, the broad (anterior) end of the egg case is positioned anteriorly within the female. Dorso‐ventral and lateral surfaces can be determined by viewing the anterior border as to whether it is visible from above (dorso‐ventral) (Figure 1) or perpendicular (lateral) to the plane of view. The basic body plan of heterodontid egg cases is similar to other oviparous elasmobranch egg cases, but with the unique feature of being twisted along its length. Egg case terminology and measurement methodology is adapted from existing egg case morphology studies (see Bustamante et al., 2013; Flammang et al., 2007; O'Neill et al., 2020) and includes novel measurements to characterize the unique morphology of heterodontid egg cases (Figure 1). The measurements taken from examined heterodontid egg cases include egg case length (ECL), anterior border width (ABW), keel width at midportion (KW1), the opposing keel width at midportion (KW2), egg case width at midportion (ECW), as seen in previous studies (e.g., White et al., 2022), and the novel measurements: keel base to adjacent keel base at midportion (KBB1), the opposing keel base to adjacent keel base at midportion (KBB2), keel base to adjacent keel apex at midportion (KBA1), and opposing keel base to adjacent keel apex at midportion (KBA2). Opposing measurements are located by rotating the egg case 180°. The number of rotations was counted by positioning the egg case dorso‐ventrally (as seen in Figure 1) and counting how many times the keel positioned at the top returns to the top position, rounded to the nearest quarter turn (Figure 1). Posterior border width (PBW) has been used in previous studies of egg case morphology but is absent or rudimentary in most heterodontid species, and damaged keels can result in erroneous measurements. Thus, this measurement was not taken for all egg cases, although it was found to be a useful character in one couplet of the dichotomous key provided. Measurements were taken to the nearest 0.01 mm using digital calipers and expressed as a proportion of ECL. All measurements are provided in Tables 1 and 2.

**TABLE 1:** Morphological characters: egg case length (ECL), egg case width at mid‐length (ECW), anterior border width (ABW), keel width midportion 1 (KW1), keel width at mid‐length 2 (KW2), keel base to adjacent keel base at midportion 1 (KBB1), keel base to adjacent keel base at midportion 2 (KBB2), keel base to adjacent keel apex at midportion 1 (KBA1), and keel base to adjacent keel apex at midportion 2 (KBA2) with mean ± SD and range (minimum–maximum) to the nearest 0.1 mm for *Heterodontus francisci* , *Heterodontus galeatus* , *Heterodontus japonicus*, and *Heterodontus mexicanus* .

Species	*Heterodontus francisci* (*n* = 7)		*Heterodontus galeatus* (*n* = 5)		*Heterodontus japonicus* (*n* = 2)		*Heterodontus mexicanus* (*n* = 4)	
Character	Mean ± SD	Range	Mean ± SD	Range	Mean ± SD	Range	Mean ± SD	Range
ECL (mm)	121.3 ± 3.00	117.2–125.6	103.9 ± 14.6	88.6–125.6	143.3 ± 1.1	142.5–144.1	80.7 ± 3.8	75.4–83.6
ABW	33.2 ± 3.4	27.5–37.9	34.3 ± 4.7	27.0–38.1	35.8 ± 1.0	35.1–36.6	Not measured	Not measured
ECW	35.8 ± 3.7	29.4–39.9	42.9 ± 6.3	35.3–50.6	40.0 ± 0.9	39.4–40.7	48.9 ± 3.5	43.4–50.9
KW1	12.2 ± 1.3	10.3–13.5	11.8 ± 1.7	9.5–13.8	10.3 ± 0.2	10.2–10.4	10.3 ± 1.6	8.5–12.0
KW2	11.9 ± 1.4	10.0–14.0	11.0 ± 1.4	9.6–13.4	10.5 ± 1.1	9.7–11.3	10.0 ± 1.7	8.4–12.0
KBA1	11.7 ± 2.3	7.8–14.3	5.7 ± 1.3	3.6–6.8	16.6 ± 0.5	16.3–17.0	12.8 ± 0.2	12.6–13.1
KBA2	11.7 ± 2.2	9.3–14.8	6.0 ± 2.4	2.2–8.0	17.3 ± 1.8	16.0–18.6	11.9 ± 0.7	11.3–12.6
KBB1	25.0 ± 3.3	21.4–29.9	15.7 ± 2.1	13.0–18.5	28.0 ± 0.4	27.7–28.2	20.7 ± 0.4	20.2–21.2
KBB2	25.8 ± 3.3	21.7–30.7	15.4 ± 2.3	11.6–17.8	28.8 ± 0.8	28.2–29.4	20.5 ± 2.6	19.1–24.4
Rotations	2.4 ± 0.1	2.25–2.5	3.2 ± 0.5	2.5–4.0	1.75 ± 0.0	1.75–1.75	2.25 ± 0.0	2.25–2.25

*Note*: Characters other than ECL are a proportion of ECL. Number of rotations to the nearest quarter turn for all egg case as a range. *n* = number of specimens.

**TABLE 2:** Morphological characters: egg case length (ECL), egg case width at midportion (ECW), anterior border width (ABW), keel width midportion 1 (KW1), keel width at midportion 2 (KW2), keel base to keel base at midportion 1 (KBB1), keel base to keel base at midportion 2 (KBB2), keel base to keel apex at midportion 1 (KBA1), and keel base to keel apex at midportion 2 (KBA2) with mean ± SD and range (minimum–maximum) to the nearest 0.1 mm for *Heterodontus portusjacksoni* and *Heterodontus zebra* and values for *Heterodontus marshallae* , *Heterodontus omanensis*, and *Heterodontus quoyi* .

Species	*Heterodontus portusjacksoni* (*n* = 5)		*Heterodontus zebra* (*n* = 3)		*Heterodontus marshallae* (*n* = 1)	*Heterodontus omanensis* (*n* = 1)	*Heterodontus quoyi* (*n* = 1)
Character	Mean ± SD	Range	Mean ± SD	Range	Values	Values	Values
ECL (mm)	129.5 ± 10.5	120.0–144.8	109.3 ± 11.6	96.6–119.2	98.5	97.3	101.7
ABW	32.7 ± 2.0	29.4–34.3	31.2 ± 3.9	27.7–35.4	41.1	26.0	28.7
ECW	39.9 ± 1.9	37.4–42.4	47.1 ± 4.6	43.4–52.2	50.7	42.8	42.9
KW1	19.7 ± 2.8	17.2–24.4	3.9 ± 0.3	3.7–4.2	4.4	3.7	9.1
KW2	19.5 ± 2.5	17.4–23.8	4.2 ± 0.4	3.9–4.6	5.3	4.0	8.3
KBA1	3.5 ± 3.0	0.0–7.5	37.9 ± 6.4	31.0–43.6	28.2	36.1	9.3
KBA2	4.1 ± 2.9	0.6–8.5	39.5 ± 6.7	32.0–44.9	30.4	36.7	8.9
KBB1	24.0 ± 1.9	22.0–26.9	39.5 ± 7.1	33.5–47.4	34.5	38.1	20.2
KBB2	24.5 ± 2.0	22.7–27.5	40.7 ± 6.3	37.0–48.0	34.1	38.6	18.8
rotations	2.7 ± 0.1	2.5–2.75	0.9 ± 0.1	0.75–1	1.5	0.75	3

*Note*: Characters other than ECL are a proportion of ECL. Number of rotations to the nearest quarter turn for all egg case as a range. *n* = number of specimens.

### 2.4 Multivariate analysis

Morphometric measurements as a proportion of ECL were subjected to non‐metric multidimensional scaling (nMDS) ordination (Clarke & Gorley, 2006). The ABW measurements were removed from the analysis because it was damaged on three of the four *H. mexicanus* egg cases. Proportional measurements were square root transformed, and a similarity matrix was constructed using the Bray–Curtis similarity coefficient. The resultant similarity matrix was analysed for differences between species using Analysis of Similarities (ANOSIM) and plotted by MDS ordination (Clarke & Warwick, 2001). The R statistic in ANOSIM is an absolute measure of differences between groups where *R* = 0 indicates no difference, and *R* = 1 indicates larger differences between groups than within groups. If the global R indicates a significant difference, pair‐wise comparisons of groups can be examined, though the significance level is influenced by sample size (replicates in a group) and must be interpreted with caution (Clarke & Gorley, 2006; Clarke & Warwick, 2001). The contribution of the individual morphometric measurements to the similarities and differences among species' egg cases was analysed using Similarity Percentage (SIMPER) (Clarke & Gorley, 2006; Clarke & Warwick, 2001). All analyses were conducted using the software PRIMER v7 (www.primer-e.com). Because the measurement ABW was removed due to several specimens having damaged borders, the above analysis was also performed with ABW included, but with the damaged specimens removed. The resulting analysis yielded similar groupings and ANOSIM results. Therefore, it was deemed that removing the ABW measurement from the analysis rather than removing three of the four specimens of *H. mexicanus* was the most appropriate.

## 3 RESULTS

### 3.1 Egg case descriptions

*Heterodontus* egg cases have a unique corkscrew shape, with pair of lateral keels spiraling its length. Egg cases are wider at the anterior end or middle and become narrower toward the posterior end. Each egg case has four respiratory fissures, each one located at the lower corners (if egg case is turned on its side) on opposing sides. Egg cases are circular (not depressed or oval) when viewed from anterior or posterior ends and tendrils (where present) are circular in cross section.

#### 3.1.1 Heterodontus francisci

**FIGURE 2:**
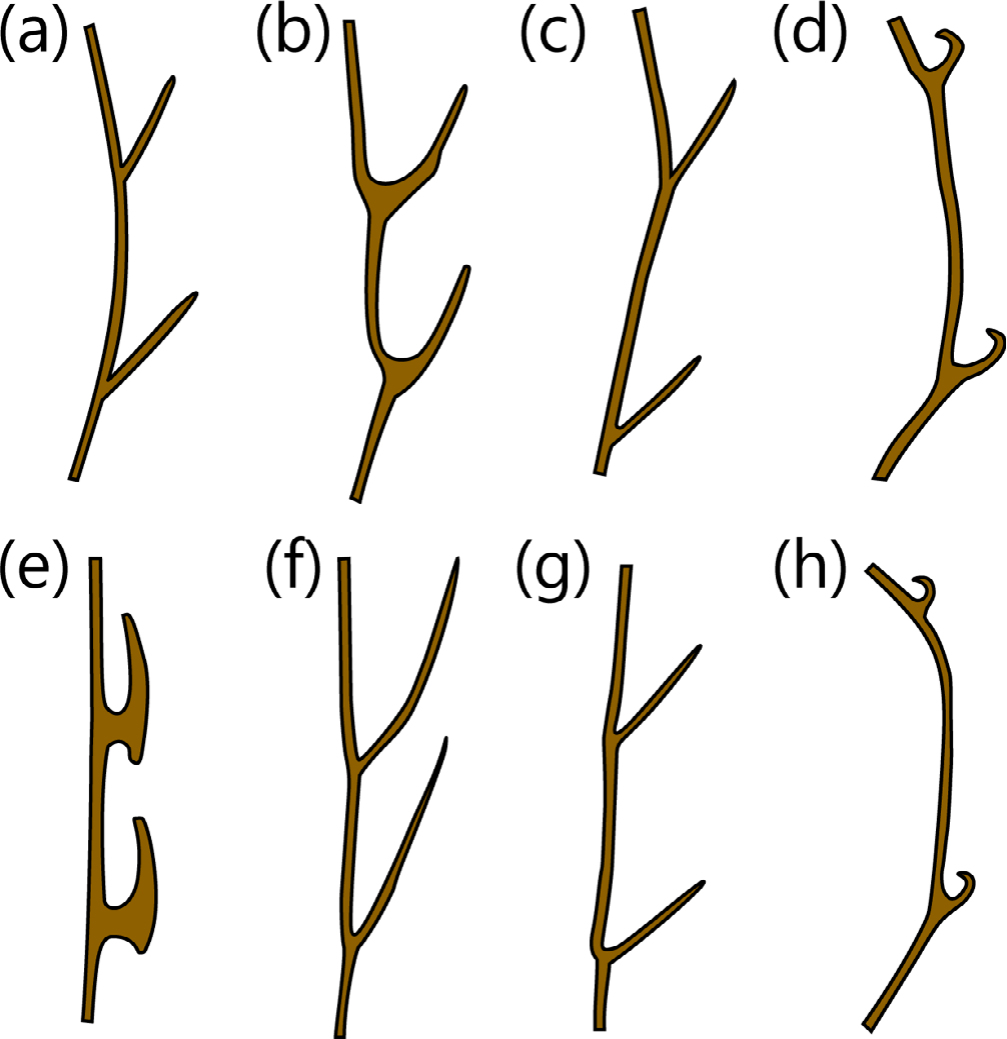
Illustration of the egg case wall and keel in cross section for (a) *Heterodontus francisci*, (b) *Heterodontus galeatus*, (c) *Heterodontus japonicus*, (d) *Heterodontus marshallae*, (e) *Heterodontus mexicanus*, (f) *Heterodontus portusjacksoni*, (g) *Heterodontus quoyi*, and (h) *Heterodontus zebra* . Each egg case is in proportion but not to scale.

**FIGURE 3:**
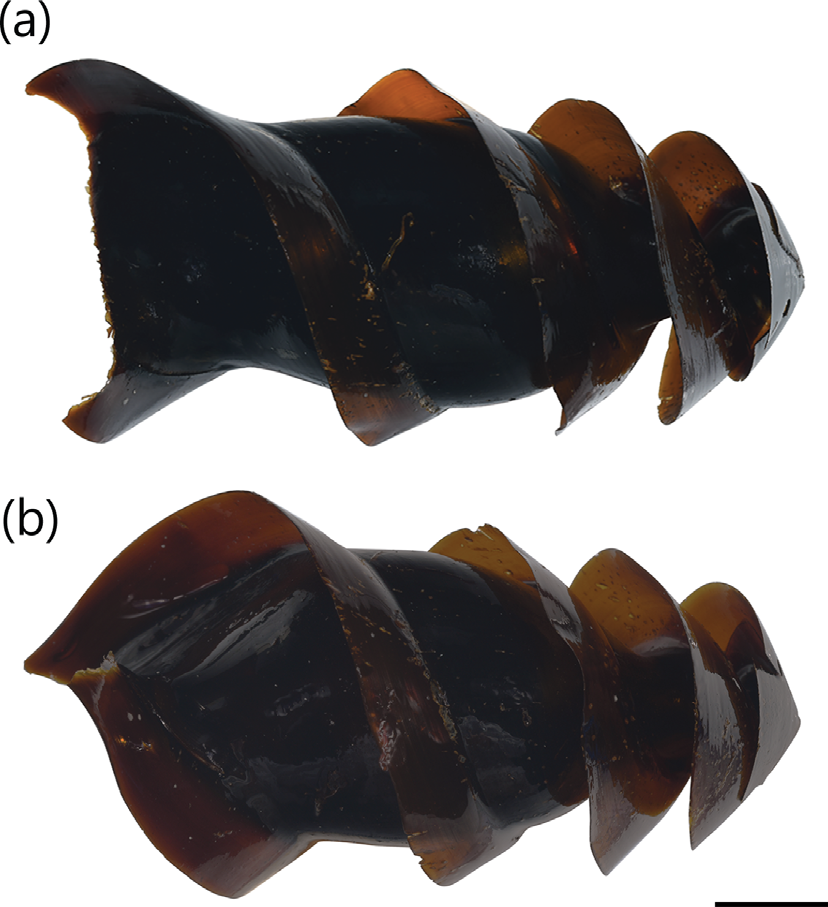
Egg case of *Heterodontus francisci* (CSIRO H 9198‐01): (a) dorso‐ventral view and (b) lateral view. Scale bar = 20 mm.

A medium‐sized heterodontid egg case (117.2–125.6 mm ECL), with lateral keels making 2¼ to 2½ rotations from anterior to posterior ends; surfaces smooth; anterior margin broad and slightly S‐shaped when viewed from anterior plane; broadest at anterior end, gradually tapering posteriorly; keel base to adjacent keel apex at midportion moderate (7.8%–14.8% ECL); keels moderate in width (10.0%–14.0% ECL), straight, projecting strongly anteriorly (Figure 2a), flexible, ending abruptly (not tapering) at posterior end. Color dark brown when preserved (Figure 3), with deposited egg cases appearing brown or with a yellowish‐brown body and brown keels.

#### 3.1.2 Heterodontus galeatus

**FIGURE 4:**
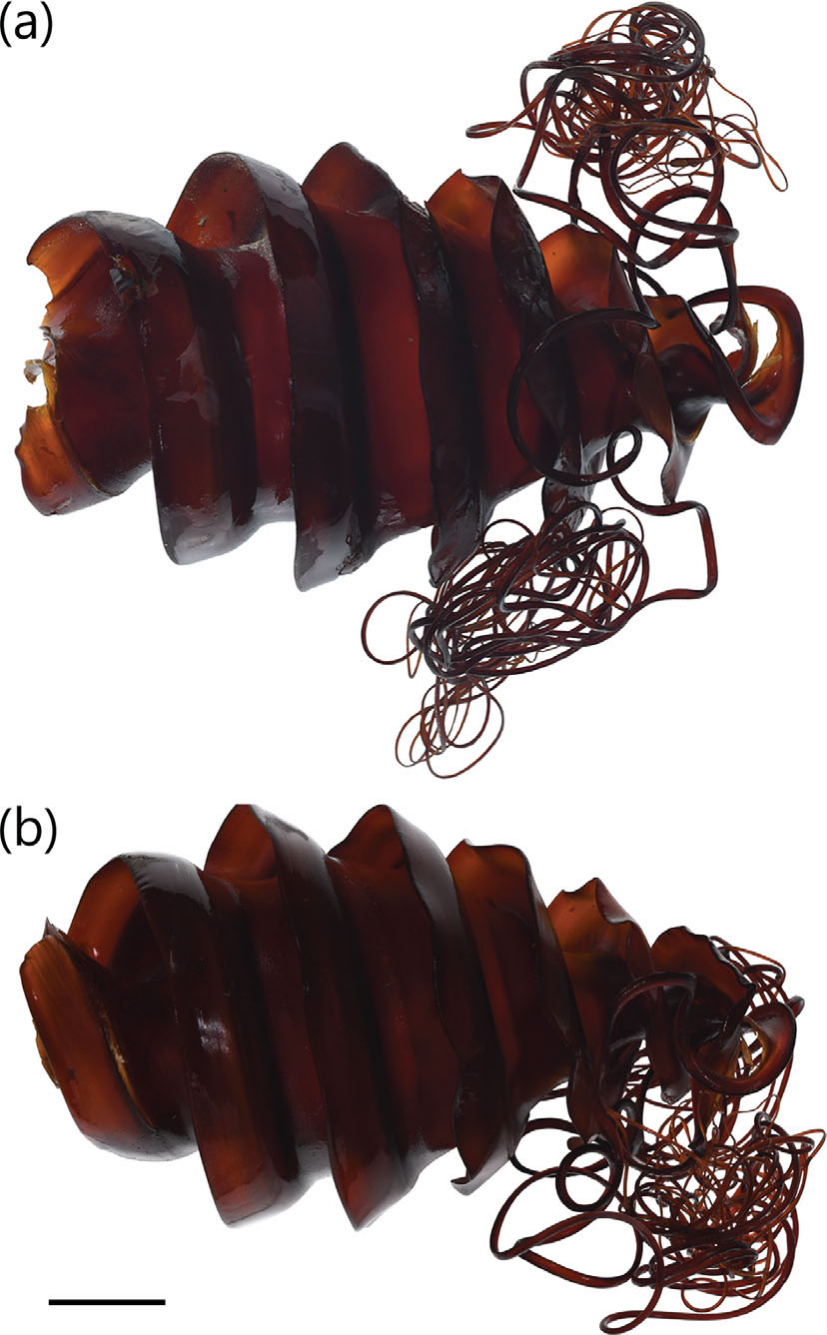
Egg case of *Heterodontus galeatus* (AMS I. 12925): (a) dorso‐ventral view and (b) lateral view. Scale bar = 20 mm.

A medium‐sized heterodontid egg case (88.6–125.6 mm ECL), lateral keels making three to four rotations from anterior to posterior ends; surfaces smooth; anterior margin broad and strongly S‐shaped when viewed from anterior plane; broadest at midportion, posterior third gradually tapering posteriorly; keel base to keel apex space at midportion small (2.2%–7.9% ECL); keels moderate in width (9.5%–13.8% ECL), firm (not flexible but not rigid), projecting anteriorly and curving anteriorly at anterior portion (Figure 2b), keels tapering into long curling tendrils at posterior end. Color reddish brown to dark brown when preserved (Figure 4), with deposited egg cases appearing reddish brown to dark brown.

#### 3.1.3 Heterodontus japonicus

**FIGURE 5:**
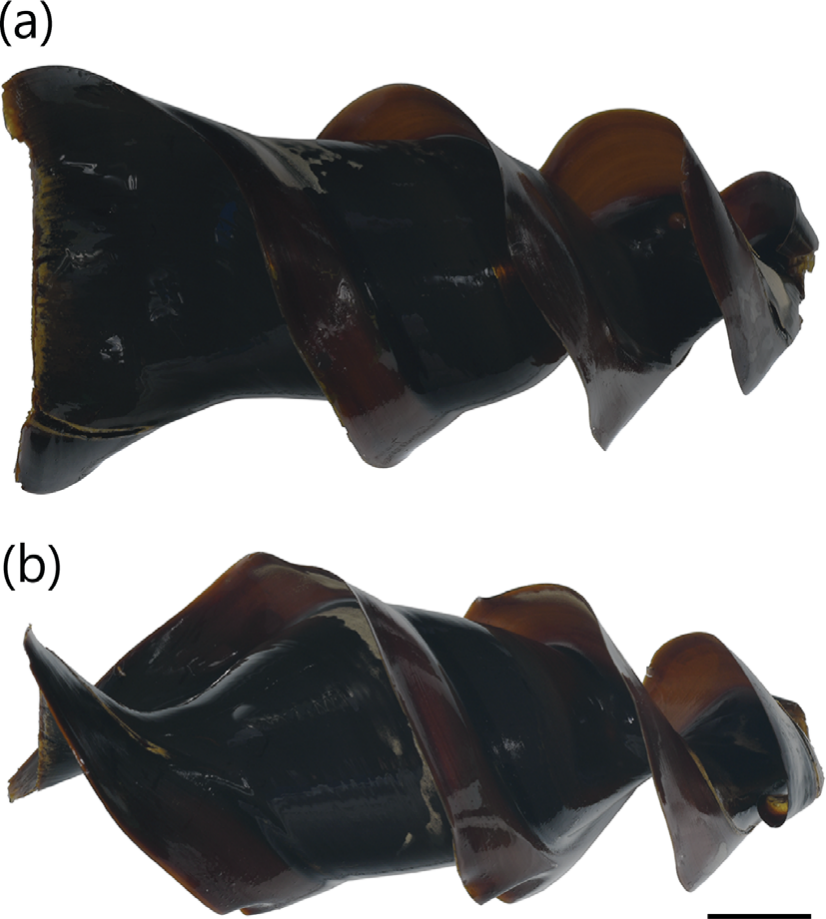
Egg case of *Heterodontus japonicus* (unregistered, from Ibaraki Prefectual Oarai Aquarium): (a) dorso‐ventral view and (b) lateral view. Scale bar = 20 mm.

A large heterodontid egg case (142.5–144.1 mm ECL), lateral keels spiraling its length making 1¾ rotations from anterior to posterior ends; surfaces smooth; anterior margin broad and weakly S‐shaped when viewed from anterior plane; widest at anterior portion, gradually tapering posteriorly from rear third; keel base to keel apex space at midportion large (16.0%–18.6% ECL); keels wide (9.7%–10.4% ECL), straight, projecting strongly anteriorly (Figure 2c), flexible, ending abruptly (not tapering) at posterior end. Color brown with slight greenish hue when preserved (Figure 5), with deposited egg cases appearing grayish brown to brown.

#### 3.1.4 Heterodontus marshallae

**FIGURE 6:**
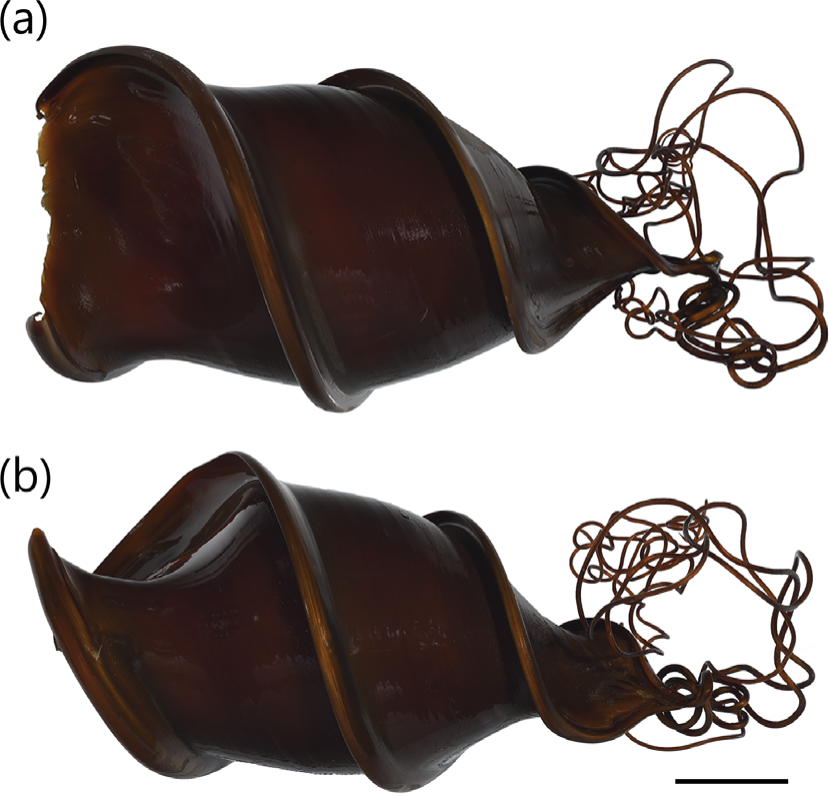
Egg case of *Heterodontus marshallae* (NTM S 18275‐001): (a) dorso‐ventral view and (b) lateral view. Scale bar = 20 mm.

Described in White et al. (2023) as a medium‐sized heterodontid egg case (98.5 mm ECL), lateral keels making 1½ rotations from anterior to posterior ends; surfaces smooth; anterior margin broad and very weakly S‐shaped when viewed from anterior plane; widest at anterior end, abruptly tapering posteriorly from posterior third; keel base to keel apex space at midportion very large (28.2%–30.4% ECL); keels narrow (4.4%–5.3% ECL), rigid, projecting outward and curving strongly anteriorly (Figure 2d), tapering into long curling tendrils at posterior end. Color golden brown with slight greenish hue when preserved (Figure 6).

#### 3.1.5 Heterodontus mexicanus

**FIGURE 7:**
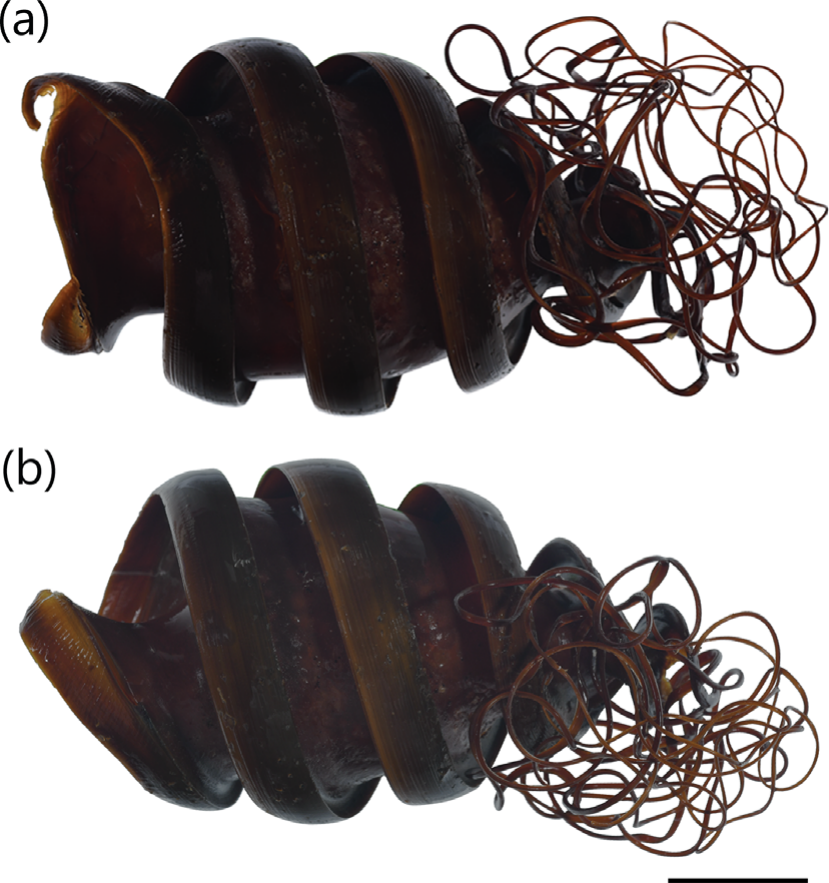
Egg case of *Heterodontus mexicanus* (CAS ICH 040578): (a) dorso‐ventral view and (b) lateral view. Scale bar = 20 mm.

A small heterodontid egg case (75.4–83.6 mm ECL), lateral keels making 2¼ rotations from anterior to posterior ends; egg case body surface smooth, keel surfaces with weak striations; anterior margin broad and slightly S‐shaped when viewed from anterior plane; widest at midportion, gradually tapering posteriorly from midportion; keel base to keel apex space at midportion moderate (11.3%–13.1% ECL); keels moderate (8.4%–12.0% ECL), thick, robust, and ridged, projecting outward from egg case body and asymmetrically T‐shaped (Figure 2e), tapering into long curling tendrils at posterior end. Color brown to golden brown when preserved (Figure 7), with deposited egg cases appearing yellowish‐brown to golden brown.

#### 3.1.6 Heterodontus omanensis

**FIGURE 8:**
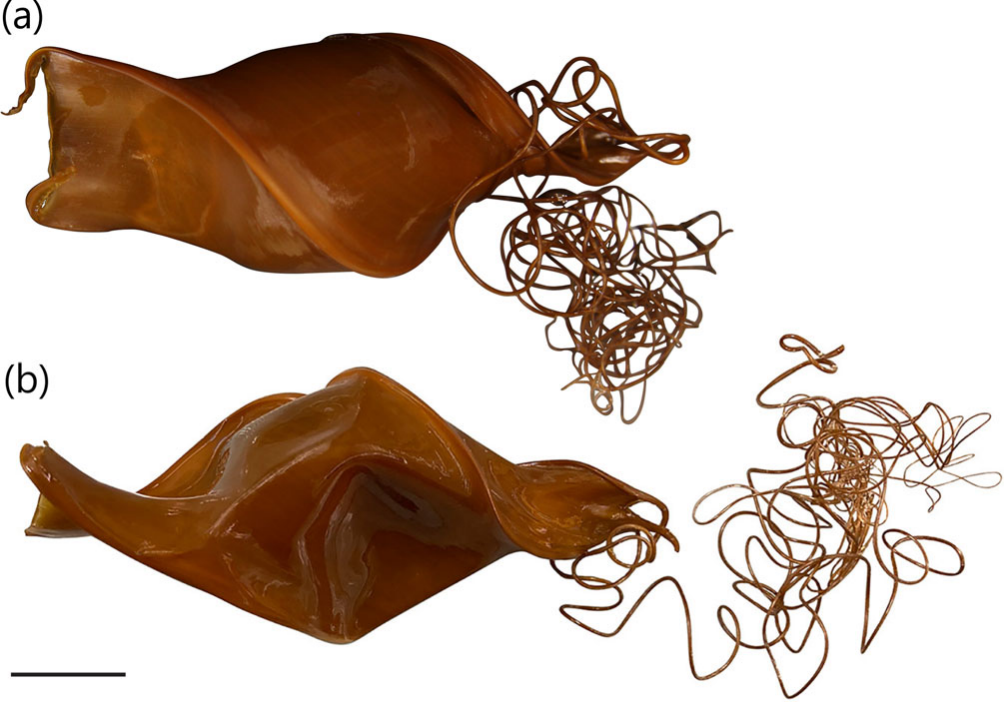
Egg case of *Heterodontus omanensis* (UW 47592): (a) dorso‐ventral view and (b) lateral view. Scale bar = 20 mm.

A medium‐sized heterodontid egg case (97.3 mm ECL), lateral keels making ¾ rotations from anterior to posterior ends; surfaces smooth; anterior margin broad slightly S‐shaped when viewed from anterior plane; widest at midportion, abruptly tapering posteriorly from midportion; keel base to keel apex at midportion very large (37.1%–37.7% ECL); keels low and rigid (3.8%–4.1% ECL), curving anteriorly, tapering into long curling tendrils at posterior end. Color golden brown when preserved (Figure 8).

#### 3.1.7 Heterodontus portusjacksoni

**FIGURE 9:**
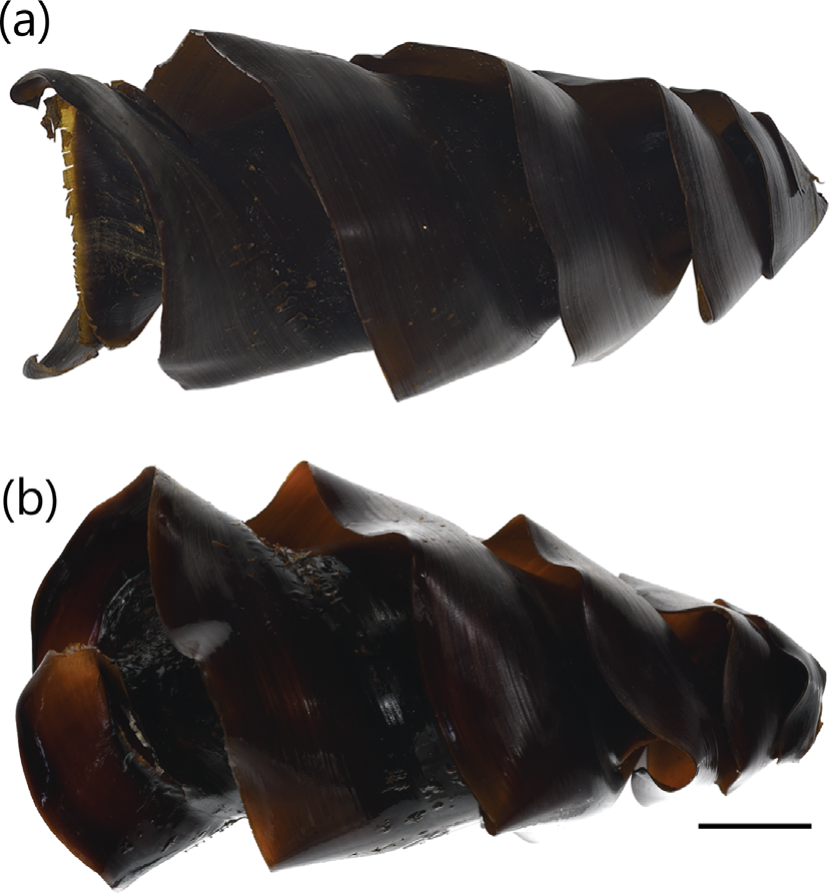
Egg case of *Heterodontus portusjacksoni* (CSIRO H 8732‐02): (a) dorso‐ventral view and (b) lateral view. Scale bar = 20 mm.

A large heterodontid egg case (120.0–144.8 mm ECL), lateral keels making 2½–2¾ rotations from anterior to posterior ends; surfaces mostly smooth; anterior margin broad and straight to weakly S‐shaped when viewed from anterior plane; widest at anterior third, slightly and gradually tapering toward posterior end; keel base to keel apex space at midportion small to keel apex abutting the base of the adjacent keel (0.0%–8.5% ECL); keels wide (16.0%–18.6% ECL), straight, projecting strongly anteriorly (Figure 2f), flexible, ending abruptly (not tapering) at posterior margin. Color dark brown to black when preserved (Figure 9), with deposited egg cases brown to dark brown.

#### 3.1.8 *Heterodontus* *quoyi*

**FIGURE 10:**
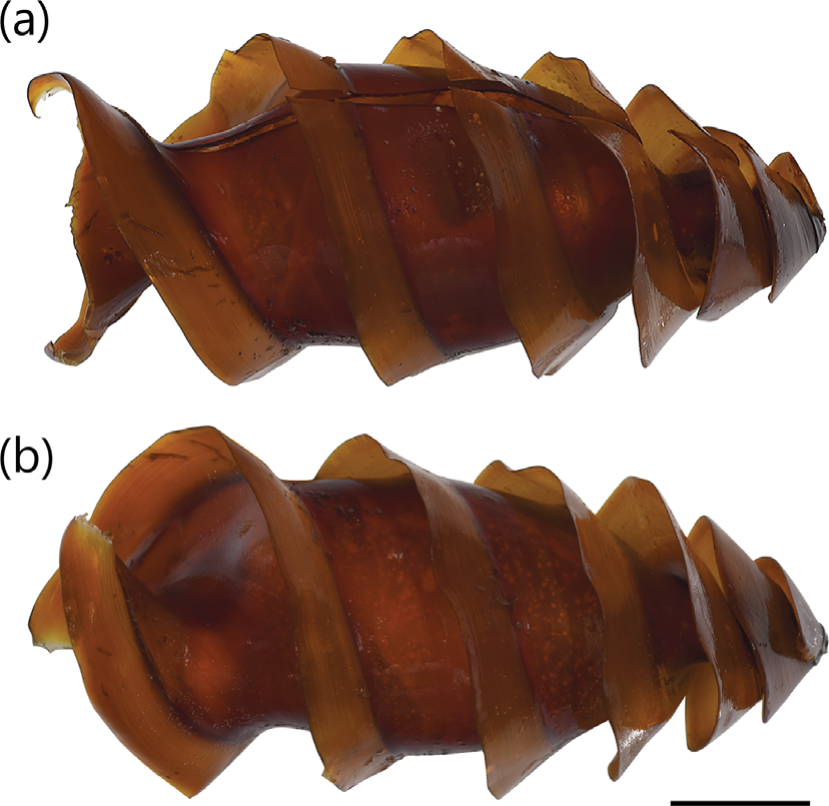
Egg case of *Heterodontus quoyi* (USNM 127779): (a) dorso‐ventral view and (b) lateral view. Scale bar = 20 mm.

A medium‐sized heterodontid egg case (101.7 mm ECL), lateral keels making three rotations from anterior to posterior ends; surfaces smooth; anterior margin broad and slightly S‐shaped when viewed from anterior plane; widest at midportion, gradually tapering posteriorly; keel base to adjacent keel apex at midportion small (8.9%–9.3% ECL); keels moderate in width (8.3%–9.1% ECL), straight, projecting strongly anteriorly (Figure 2g), flexible, ending abruptly (not tapering) at posterior margin. Color light golden brown when preserved (Figure 10).

#### 3.1.9 Heterodontus zebra

**FIGURE 11:**
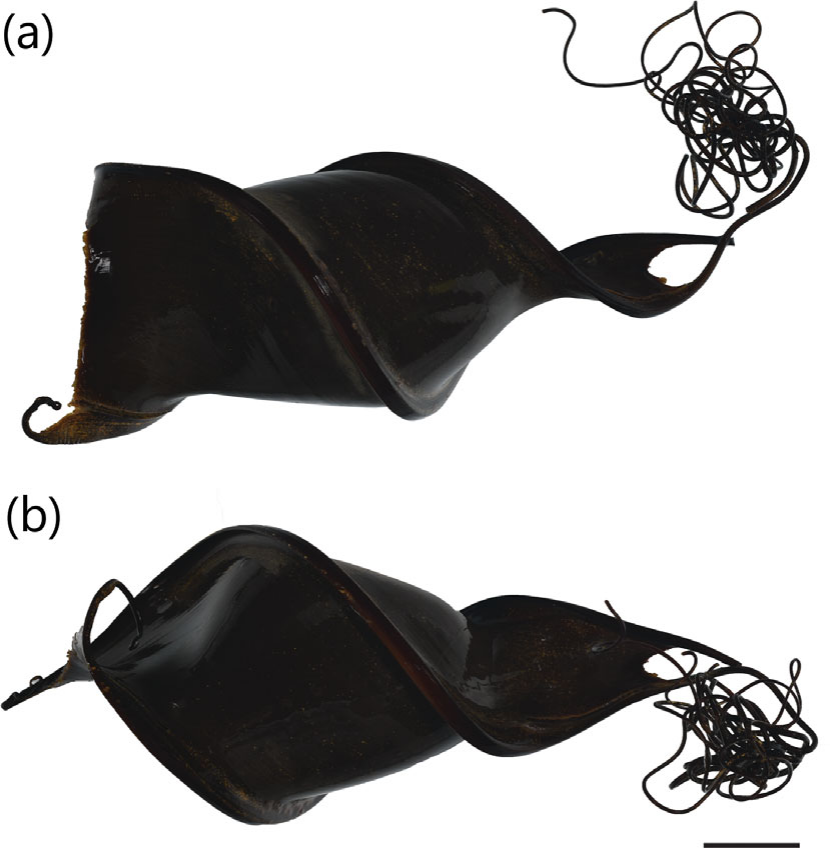
Egg case of *Heterodontus zebra* (unregistered, from Ibaraki Prefectual Oarai Aquarium): (a) dorso‐ventral view and (b) lateral view. Scale bar = 20 mm.

A medium‐sized heterodontid egg case (96.6–119.2 mm ECL), lateral keels making 0.75–1 rotation from anterior to posterior ends; surfaces smooth; anterior margin broad, weakly S‐shaped when viewed from anterior plane; widest at anterior half, abruptly tapering at posterior third; keel base to keel apex space at midportion very large (31.0%–44.9% ECL); keels narrow (3.7%–4.6% ECL), rigid, projecting slightly outward and curving strongly anteriorly (Figure 2h), tapering into long curling tendrils at posterior end. Color olive‐greenish brown when fresh and light golden brown, or dark brown to black when preserved (Figure 11).

### 3.2 Multivariate analysis of egg case morphology

**FIGURE 12:**
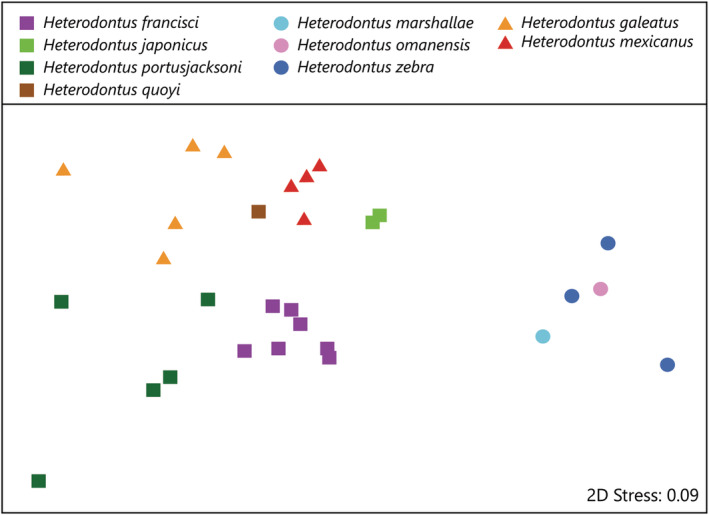
Non‐metric multidimensional scaling (MDS) ordination plot of the 29 egg cases based on Bray–Curtis similarity between proportional measurements from *Heterodontus francisci* , *Heterodontus galeatus* , *Heterodontus japonicus* , *Heterodontus marshallae* , *Heterodontus mexicanus* , *Heterodontus omanensis*, *Heterodontus portusjacksoni*, *Heterodontus quoyi* , and *Heterodontus zebra* . Square symbols denote egg cases with “wide keeled lacking tendrils” morphotype; circle symbols denote egg cases with “narrow keeled with tendrils” morphotype, and triangle symbols denote “wide keeled with tendrils” morphotype.

**FIGURE 13:**
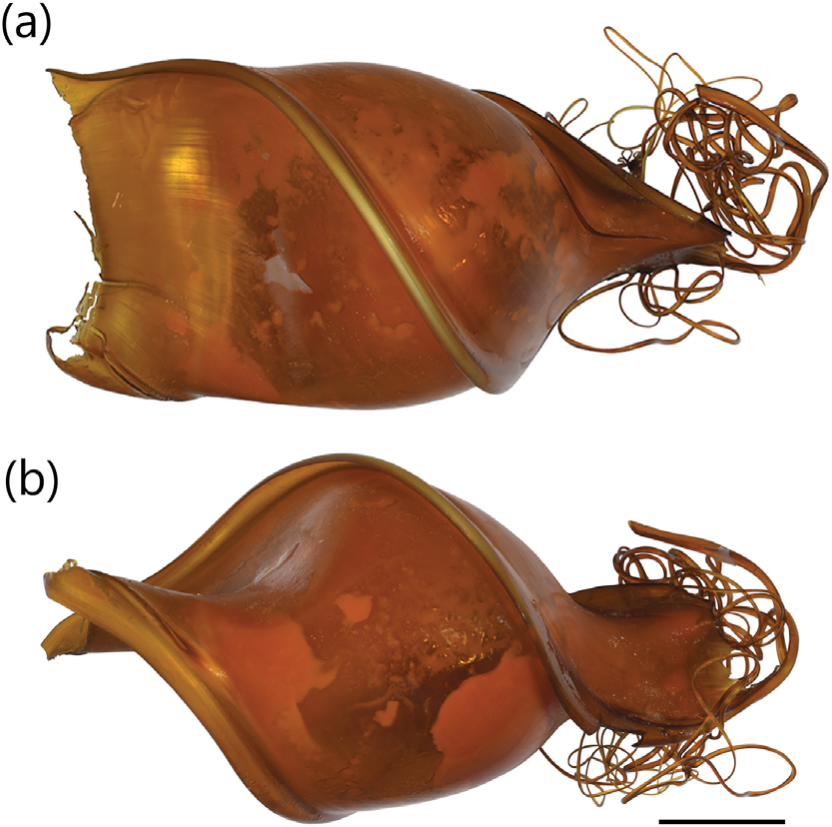
Egg case of *Heterodontus zebra* (KAUM‐I. 69456): (a) dorso‐ventral view and (b) lateral view. Scale bar = 20 mm.

Non‐metric MDS ordination of egg case morphometric characters of different species of *Heterodontus* shows clear grouping with only the *H. omanensis* and *H. zebra* samples overlapping (Figure 12). All egg cases with wider keels form a distinct grouping to the left of the plot, and all egg cases with narrow keels from a distinct grouping to the right of the plot. ANOSIM showed the egg case morphometrics of the nine species were significantly different overall (*p* < 0.01; *R* ^2^ = 0.79) and in the following pair‐wise combinations: *H. japonicus* versus *H. galeatus* and *H. portusjacksoni* (*p* < 0.05; *R* ^2^ = 0.86–1); *H. mexicanus* versus *H. galeatus*, *H. francisci*, *H. portusjacksoni*, and *H. zebra* (*p* < 0.05; *R* ^2^ = 0.52–1); *H. galeatus* versus *H. francisci*, *H. portusjacksoni*, and *H. zebra* (*p* < 0.05; *R* ^0^ = 0.71–1); *H. francisci* versus *H. portusjacksoni* and *H. zebra* (*p* < 0.05; *R* ^2^ = 0.85–1); *H. portusjacksoni* versus *H. zebra* (*p* < 0.05; *R* ^2^ = 1). All other pair‐wise combinations were not significant (*p* > 0.05) primarily due to reduced sample sizes, that is, *H. marshallae*, *H. omanensis*, and *H. quoyi* represented by a single egg case only.Intraspecific differences were low with an average SIMPER similarity above 93.7% for all species where more than one egg case was measured. Despite these results, *H. zebra* exhibited some intraspecific variation, with one egg case (KAUM I.69456) from the Philippines (Figure 13) appearing shorter in length, broader in width, and having greater distances between keels, that is, keel base to adjacent keel base (KBB) and keel base to adjacent keel apex at midportion (KBA), compared to the other *H. zebra* egg cases, which were captive bred at Ibaraki Prefectual Oarai Aquarium.

SIMPER analysis indicated that keel base to adjacent keel apex at mid‐portion (KBA) is most responsible for all pair‐wise comparisons between species' egg cases, except for comparisons between *H. mexicanus* and *H. francisci* and *H. portusjacksoni* and *H. galeatus*. In *H. galeatus* and *H. francisci* keel base to adjacent KBA was the second top contributor to the differences between the two species after keel base to adjacent KBB. Egg case width at midportion (ECW) is most responsible for differences between *H. mexicanus* and *H. francisci*, whereas keel width at midportion (KW1 and KW2) is most responsible for differences between *H. portusjacksoni* and *H. galeatus*.

### 3.3 Key to the egg cases of *Heterodontus* species

1. Long tendrils extending from posterior (narrow) end of egg case …………………… 2.

No tendrils on egg case ………………………………………………………… 6.

2. Lateral keels T‐shaped in cross section …… *H. mexicanus* (eastern Pacific).

Lateral keels not T‐shaped in cross section …………………………… 3.

3. Lateral keels broad, keel width >9% ECL………………….………….……………………………………………………. *H. galeatus* (southwestern Pacific).

Lateral keels very narrow <6% ECL ……………….……………………… 4.

4. Egg case with 1½ rotations; anterior border width ~ 41% ECL…………………………………………. *H. marshallae* (eastern Indian Ocean).

Egg case with ¾–1 rotation; anterior border width <36% ECL ……………………………………………………………………………………………… 5.

5. Posterior border present, its width 3% ECL; only found in the Western Indian Ocean ……………………………………*H. omanensis* (western Indian Ocean).

Posterior border absent, its width not measurable; only found in the northwest Pacific ……………………………. *H. zebra* (northwest Pacific).

6. Egg case small, ~100 mm ECL ……… *H. quoyi* (eastern Pacific).

Egg case large, >115 mm ECL …………………………………………………7.

7. Posterior border present, its width >10% ECL. …………………………………………………………………….…………. *H. japonicus* (northwest Pacific).

Posterior border absent……………………………………………………………8.

8. Keels large, their width 17%–25% ECL …………………………………… …………….…………………………… *H. portusjacksoni* (southwestern Pacific).

Keels much smaller, their width 10%–14% ECL *…*……………………………………..……………………*H. francisci* (eastern Pacific).

## 4 DISCUSSION

### 4.1 Morphological differences

The egg cases of some heterodontid sharks are relatively well known; for example *H. portusjacksoni* and *H. francisci* are well studied compared to other heterodontids due to their distinctive and easily identifiable shape, prevalence, and ease of accessibility in shallower waters. The placid nature of heterodontid sharks also makes them a good subject for study (Powter & Gladstone, 2008a, 2008b; Strong 1989). The remaining species are poorly studied due to a lack of specimens in biological collections, the difficulty obtaining or observing specimens because of their occurrence in greater depths making them harder to sample, and some being rarely encountered. For example, *H. marshallae* was only described very recently (2023) based on the limited material, which fortunately included an egg case. Similarly, *H. omanensis* is only known from a small number of specimens, but luckily the holotype was a gravid female, and the egg case had been removed and retained when it was described by Baldwin (2005). This paper provides thorough descriptions, morphometrics, and comparisons of the egg cases of 9 of the 10 nominal heterodontid species and makes notes of their biology and oviposition.

Heterodontid egg cases are unique and easily distinguishable from those of other sharks by their spiraled or “corkscrew” shape. Fischer et al. (2014) separated oviparous egg cases into different morphotypes, listing *Heterodontus* as unique from other egg case morphotypes, that is, Scyliorhinidae, Rajidae, and Orectolobidae. Although distinct, the basic body plan and features of heterodontid egg cases share similarities with other elasmobranch egg cases, such as the anterior border width where the embryo exits the egg case, a narrower posterior end where the egg case emerges from the female, lateral keels, and tendrils (Figure 1), but have the unique feature of being twisted along its length.

The diversity of chondrichthyan egg cases has resulted in the generation of many suites of measurements, for example, for Scyliorhinidae, see Ebert et al. (2006); for *Bathyraja*, see White et al. (2022); for *Parascyllium*, see Caruso and Bor (2007), and for chimaeras, see Mancusi et al. (2021). The unique structure of *Heterodontus* egg cases, combined with previous variations in descriptions and meristic results (e.g., Baldwin [2005]; Waite [1896]), calls for the creation of a new suite of bespoke morphological and meristic measurements. This study aims to standardize the terminology and methodology for measuring *Heterodontus* egg cases.

### 4.2 Interspecific differences

Some chondrichthyan egg cases have been separated into different morphotypes at genus level. For example, *Apristurus* egg cases can be separated into morphotypes (Flammang et al., 2007), which reflect established species groupings, for example *brunneus* group, *longicephalus* group, and *spongiceps* group (Compagno, 1988). *Heterodontus* egg cases in this study can also be separated into different morphotypes: the “wide keeled lacking tendrils” form, which includes *H. francisci*, *H. japonicus*, *H. portusjacksoni*, and *H. quoyi*; the “narrow keeled with tendrils” form, which includes *H. marshallae*, *H. omanensis*, and *H. zebra*; and the “wide keeled with tendrils” form, which includes *H. galeatus* and *H. mexicanus*. The “narrow keeled with tendrils” egg cases form a distinct out‐group on the nMDS (Figure 12). The distinction between the other groups “wide keeled with tendrils” and “wide keeled lacking tendrils” is less clear based on the nMDS plots (Figure 12). This could be because the measured characters used in the nMDS aren't different enough to distinguish the two groups, and the key features for distinguishing these groups (the presence/absence of tendrils) are not accounted for in the nMDS analysis. Waite (1896) recorded that a key difference between the egg cases of *H. portusjacksoni* and *H. galeatus* is the latter species' long tendrils.

Another feature not accounted for in the nMDS analysis is the T‐shaped keels belonging to *H. mexicanus*, which is a key identifying feature of this species, as all other heterodontid egg cases have small curved, or wide, flat keels that are thin. *H. mexicanus* is also the smallest of the heterodontid egg cases, ranging from 75.4 to 83.6 mm ECL, versus other egg cases, which range from 88.6 to 144.8 mm ECL. *H. galeatus* is similar to *H. mexicanus* in having large keels and tendrils, but the keels of *H. galeatus* are thin (vs. T‐shaped in *H. mexicanus*). *H. galeatus* is distinguishable from other species by having large, wide keels that are thin and long tendrils extending from the posterior end (vs. wide keels and no tendrils or narrow keels and the presence of tendrils).

*Heterodontus francisci*, *H. japonicus*, *H. portusjacksoni*, and *H. quoyi* differ from *H. marshallae*, *H. omanensis*, and *H. zebra* in not having tendrils (vs. tendrils present) and differences in keel width and keel positioning. Specifically, *H. francisci*, *H. japonicus*, *H. portusjacksoni*, and *H. quoyi* have larger keel width (KW) measurements, smaller keel base to adjacent keel apex at mid‐portion (KBA) and smaller keel base to adjacent keel base at mid‐portion (KBB) measurements than *H. marshallae*, *H. omanensis*, and *H. zebra*. In addition, *H. portusjacksoni* and *H. francisci* have a greater ECL than *H. marshallae, H. omanensis*, and *H. zebra*.

*Heterodontus francisci* differs from *H. japonicus* by having a greater ECL (vs. smaller) and smaller keel base to adjacent keel apex at mid‐portion (KBA) measurements (vs. larger). *H. francisci* differs from *H. portusjacksoni* by having a smaller KW (vs. larger) and from *H. quoyi* by having a larger ECL (vs. smaller).

*Heterodontus japonicus* differs from *H. portusjacksoni* by having a larger KW (vs. smaller) and larger keel base to adjacent keel apex at midportion (KBA) measurements (vs. smaller). *H. japonicus* differs from *H. quoyi* by having a larger ECL (vs. smaller), larger keel base to adjacent keel apex at mid‐portion (KBA), and keel base to adjacent keel base at mid‐portion (KBB) measurements (vs. smaller). *H. portusjacksoni* differs from *H. quoyi* by having a larger ECL (vs. smaller) and larger KW (vs. smaller).

*Heterodontus marshallae*, *H. omanensis*, and *H. zebra* are similar in having narrow keels and tendrils from their posterior end. *Heterodontus marshallae* differs from *H. zebra* and *H. omanensis* in having a larger anterior border width (ABW) (41.1% ECL for *H. marshallae* vs. 27.7–35.4% ECL and 26.0% ECL for *H. zebra* and *H. omanensis* respecively) and number of rotations (1½ rotations for *H. marshallae* vs. ¾–1 and ¾ rotations for *H. zebra and H. omanensis,* respectively). *Heterodontus omanensis* differs from *H. zebra* by having a posterior border width (PBW) present compared to absent and only occurring in the Western Indian Ocean (vs. only occurring in the northwest Pacific). It should be noted that only limited specimens of *H. japonicus*, *H. marshallae*, *H. omanensis*, and *H. quoyi* were available for study, and these measurements should be interpreted with caution.

### 4.3 Intraspecific differences

Overall intraspecific differences of egg case morphology were low; however, some features did display high variability in some species, such as ECL for *H. portusjacksoni*, *H. zebra*, and *H. galeatus* which ranged between 120.0 and 144.8 mm, 96.6 and 119.2 mm, and 88.6 and 125.6 mm, respectively. Egg case size is believed to be related to female size, with larger females producing larger egg cases (D. Moreno, University of Tasmania, personal communication). Investigations using captively held specimens could be performed to better understand this relationship.

In this study, the number of keel rotations on *H. portusjacksoni* egg cases ranged from 2½ to 2¾, whereas McLaughlin and O'Gower (1971) reported a slightly greater number of turns (by ¼ turn more), ranging between 2½ and 3 rotations. McLaughlin and O'Gower (1971) had a much larger sample size of egg cases (>43), which encompassed a greater range of variation. Waite (1896) documented 5 or 6 turns when describing the egg cases of *H. galeatus* and *H. portusjacksoni*.

When describing *H. omanensis*, Baldwin (2005) counted the number of turns made by the keels as 2, which has later been used in Ebert et al. (2021). It is not clear how the turns were counted as the number of full rotations counted in this study was ¾ for this species. This variation can be accounted for by differences in methodology, as the egg case counted by Baldwin (UW 47592) was the same as the one used in this study. More egg cases of this species would allow for a more in‐depth assessment to understand intraspecific variation.

Waite (1896) documented the egg cases of *H. galeatus* and *H. portusjacksoni* to “…together make five or six turns…,” whereas in this study rotations were 2.5–4.0 and 2.5–2.75, respectively. This is likely due to differences in methodology, as no standard had been developed at this point, and it exceeds the counts of both species within this study.

Egg case specimen KAUM I.69456 from the Philippines has been identified as *H. zebra* based on its location within the known distribution of *H. zebra* and its morphology resembling other *H. zebra* egg cases. After further investigation and comparison to captive‐laid *H. zebra* egg cases, it was noted that KAUM I.69456 is shorter in length, broader in width, and has greater distances between keels, that is, keel base to adjacent keel base at midportion (KBB) and keel base to adjacent keel apex at midportion (KBA) measurements, compared to the captive‐laid *H. zebra* (see Figures 11 and 13). The difference in shape could be attributed to two of the specimens being captive‐bred versus wild, or it could be natural variation across its range. A small sample size (three) makes it difficult to draw conclusions about the cause for this variation, and more specimens of this species' egg cases should be kept for further investigation.

### 4.4 Ecological information

**FIGURE 14:**
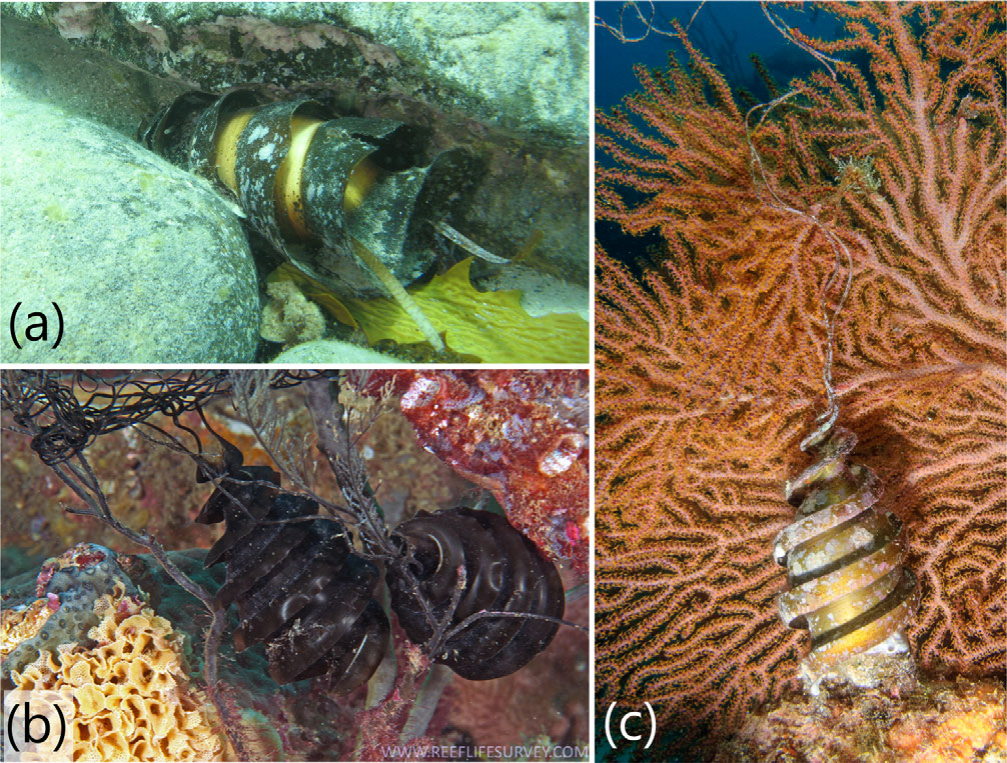
Underwater images of wild *Heterodontus* egg cases: (a) *Heterodontus portusjacksoni* (image by ©kittsw, iNaturalist https://www.inaturalist.org/observations/105988912) wedged between boulders, Second Valley, South Australia edited from owner (CC BY‐NC); (b) *Heterodontus galeatus* (image by Ian Shaw—Reef Life Survey, CC BY 3.0 https://en.wikipedia.org/wiki/Crested_bullhead_shark#/media/File:Heterodontus_galeatus_egg_case.jpg) attached to coral substrate, North Solitary Island, New South Wales, Australia, and (c) *Heterodontus mexicanus* (image by Andy Murch, sharksandrays.com) with tendrils attached to sea fan.

Like the egg cases of other oviparous species, the egg cases of heterodontids have evolved different adaptations to optimize successful embryonic development and reduce mortality. The evolution of different anchorage strategies, including large keels, long tendrils, and thickened walls, is effective in reducing mechanical shock from wave action and preventing egg cases from being transported away from the deposition site to less‐desirable areas, such as those with increased exposure to predation, reduced oxygen levels, or even washed ashore (Vazquez et al., 2018; Waite, 1896). Female *H. portusjacksoni* are found on sandy bottoms adjacent to rocky reefs and use specific deposition sites commonly at depths of <15 m, where they have been observed depositing their egg cases directly into rock crevices (Ebert et al., 2021; McLaughlin & O'Gower, 1971). Two egg cases are laid every 8–17 days during August–September. Once deposited, tidal surges, currents, and swell move the egg case deeper into rock cervices, where their corkscrew shape helps wedge and anchor the egg case in place (Figure 14a) (Wildlife Instincts: Australia's Curious Port Jackson Sharks, n.d.). The egg cases become so well lodged that Waite (1896) stated they “are so jammed…they can only be removed either by turning them round and withdrawing them small end first, or by actually unscrewing them.” This process prevents the egg case from being dislodged and swept away and helps keep the egg case out of reach from common predators: *H. galeatus*, common tent shell *Astralium tentoriformis*, and other large predatory fish (Powter & Gladstone, 2008a). Incubation time is about 12 months and young hatch at ~23 cm TL.

*Heterodontus francisci* occupy rocky, kelp covered reefs with sand gullies in the intertidal zone mostly between 2 and 11 m, but as deep as 150 m. Displays short single oviparity, laying a pair of egg cases every 11–14 days during February to April in captivity (Ebert et al., 2021). In the wild, *H. francisci* have been observed to wedge egg cases between or beneath rocks (Barnhart, 1932). In captivity egg cases have been observed deposited on sandy bottoms and sometimes later cannibalized by adults (Compagno, 2001). Incubation time is 7–9 months, and young hatch at 15–17 cm TL.

*H. japonicus* is predominantly a coastal species (<40 m depth), but sometimes occurs to depths of 100 m, primarily occurring on rocky, kelp‐covered reefs (Ebert et al., 2021). Displays short single oviparity, laying egg cases in pairs in shallow waters (8–9 m) during March to September. Incubation time is about 12 months and young hatch at 18 cm TL. Female *H. japonicus* and *H. portusjacksoni* (and probably other “wide keeled lacking tendrils” species, that is, *H. francisci* and *H. quoyi*), lay egg cases in selected deposition sites creating “nests,” where up to 15 egg cases at different stages of development have been observed (Smith, 1942).

*H. quoyi* is also a coastal species occurring in water <40 m depth on rocky reef, coral reef, and sand (Ebert et al., 2021). Little else is known about its biology; its egg case morphology suggests it deposits its egg cases in similar habitats and fashion to the other “wide keeled lacking tendrils” species, that is, among rock crevices where the egg cases become lodged, but more investigation is needed to confirm this.

*H. galeatus* is mostly found in coastal waters inhabiting rocky reefs, seagrass beds, and seaweed (Ebert et al., 2021), but its distribution also extends onto the continental shelf to depths of at least 93 m deep. Displays short single oviparity, laying egg cases in pairs during July and August in 20–30 m by attaching their tendrils to seaweed or sessile macroinvertebrates such as sponges (Last & Stevens, 2009; Waite, 1896) (Figure 14b). Incubation time is short for this species, lasting 5–8 months. Young hatch at about 22 cm TL.

*H. mexicanus* also occurs in coastal waters, to depths of 50 m, on rocky and coral reefs on sandy bottoms, and around shallow seamounts (Ebert et al., 2021). Egg case tendrils attach to kelp or sessile macroinvertebrates such as coral and sponges (Figure 14c). Compagno (2001) suggests that *H. mexicanus* has thick T‐shaped keels to protect the egg case from mechanical shock from waves and currents, and to protect against predation as opposed to anchorage, which is provided by its tendrils. Young hatch at ~14 cm TL. Little else is known about the reproductive output of this species.

*H. zebra* occurs mostly in coastal waters to depths of at least 50 m. In the wild, egg cases are likely attached by their long posterior tendrils to sessile macroinvertebrates, such as coral or sponges, or to seaweed. Observations of captive *H. zebra* depositing egg cases demonstrated that they emerged tendrils first from the females' cloaca. The tendrils were wrapped around decorative corals or rocks by the female swimming in circles. Displays short single oviparity, laying a pair of egg cases each laying event. The first egg case took approximately 2.5 h to deposit; the second took approximately 1 h to deposit. Laying of egg cases by captive *H. zebra* occurred across two separate occasions 6 months apart. The frequency of laying consisted of 10 egg cases, at 12–18‐day intervals between February and April, and four egg cases at a 17‐day interval, between November and December. Incubation lasted about 5 months, and the young were hatched at 15–17 cm TL.

*H. marshallae* occurs on the outer continental shelf and upper continental slope at depths of 125–230 m (White et al., 2023). *H. omanensis* occurs in tropical waters at depths of 72 m; very little is known about the biology of these two species. The egg cases of *H. marshallae* and *H. omanensis* have not been observed in the wild, but based on their similar morphology to *H. zebra*, it is assumed that they attach their egg cases in a similar fashion, attaching their egg case to sessile macroinvertebrates or other structure by their long tendrils.

Species with narrow keels and tendrils (*H. marshallae*, *H. omanensis*, and *H. zebra*) are restricted to the Indo‐west Pacific, whereas egg cases with large keels are found throughout the western and eastern Pacific. *H. galeatus* and *H. portusjacksoni* are sympatric with an overlapping distribution ranging from southern Queensland to southern New South Wales. The egg case of *H. galeatus* has medium‐to‐large‐sized keels and long tendrils extending from its posterior end, versus large keels and lacking tendrils in *H. portusjacksoni*. These species have different behaviors of egg case deposition, with *H. galeatus* laying its egg cases at greater depths (20–30 m) attaching them to benthic invertebrates such as sponges or seaweed, whereas *H. portusjacksoni* deposits its egg cases in rocky crevices between rocks at shallower depths of <15 m (McLaughlin & O'Gower, 1971). Predation is a major source of mortality of elasmobranch egg cases with estimates ranging from 14% to 42% for Atlantic skate species (Lucifora & García, 2004); 22% *Leucoraja erinacea* (Cox & Koob, 1993); 14%–40% for coastal elasmobranchs from South Africa (Smith & Griffiths, 1997); 18% for *Amblyraja radiata* (Cox et al., 1999). Estimated mortality rates of *H. portusjacksoni* egg cases are as high as 89.1% and of those that did not survive, 97.7% were the result of predation primarily by large predatory fish and including *H. galeatus* (Powter & Gladstone, 2008a). Powter and Gladstone (2008a) reported very low predation rates by gastropods compared to other studies on other oviparous species (see above). Powter and Gladstone (2008a) theorize that *H. portusjacksoni* egg cases suffer low predation by gastropods because they are elevated above the seafloor, wedged between rocks, and not in contact with sand substrate making it harder for gastropods to access them, a theory supported by the investigations of Cox and Koob (1993). The different morphology and oviposition of *H. portusjacksoni* and *H. galeatus* egg cases could be a niche partitioning strategy to increase survivability of their respective young.

This niche partitioning theory could also apply to *H. mexicanus* that has an overlapping distribution with *H. francisci* and *H. quoyi* off California and Mexico in the northeastern Pacific. Like *H. galeatus* and *H. portusjacksoni, H. mexicanus* differs in its morphology by having robust T‐shaped keels and tendrils, compared to *H. francisci* and *H. quoyi* that has wide keels and lack tendrils. More research is needed to investigate this niche partitioning theory.

### 4.5 *Heterodontus portusjacksoni* range clarification

**FIGURE 15:**
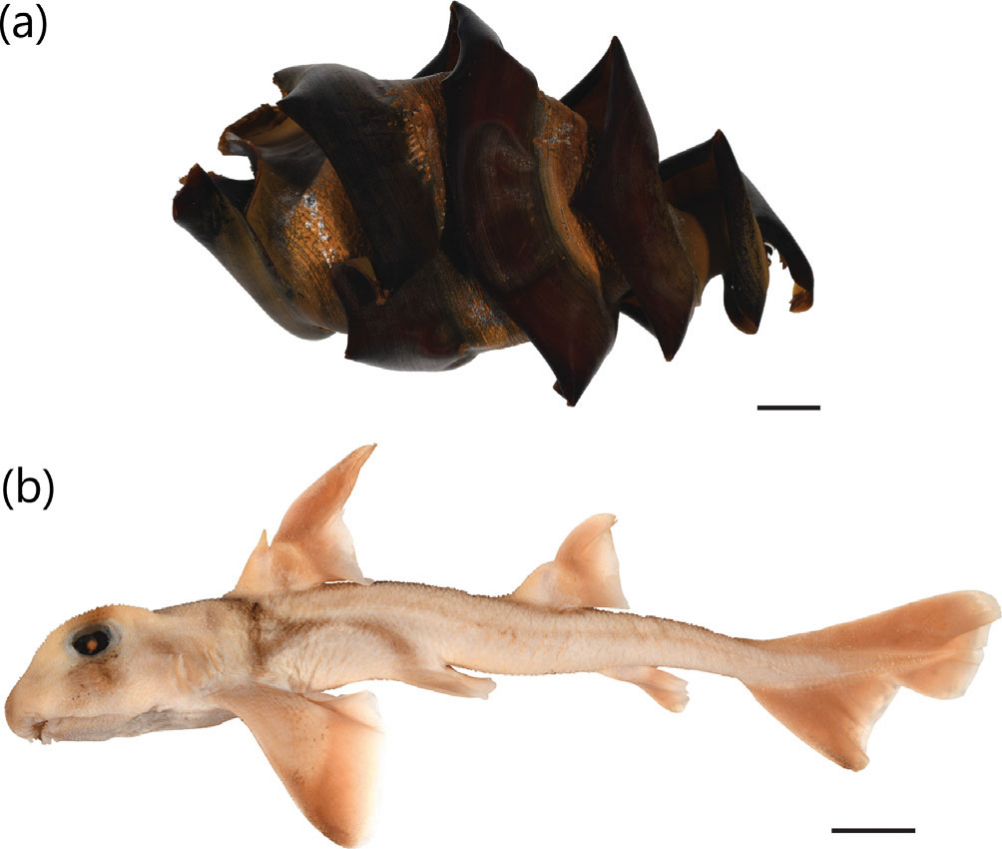
*Heterodontus portusjacksoni* specimens from York Sound, Western Australia: (a) egg case BMNH 1920.10.11.1 and (b) 234 mm TL (total length) female neonate NTM S.00043‐001. Scale bar = 20 mm.

*H. portusjacksoni* is known to occur across southern Australia, from Byron Bay (New South Wales) to Houtman Abrolhos (Western Australia), including Tasmania. Several questionable records for this species have existed outside of this range, including off Broome and York Sound in northern Western Australia and Moreton Bay in southeast Queensland. The Western Australian records are based on two specimens: a dried egg case specimen BMNH 1920.10.11.1 collected from near Broome (misspelt “Brrom” on specimen label), originally identified as *Cestracion* (Figure 15a) and a 234 mm TL female neonate NTM S.00043‐001 from York Sound (Figure 15b). Both specimens were examined in this study and can be confirmed as *H. portusjacksoni*. These records suggest a substantial northward extension of ~2000 km from Houtman Abrolhos to York Sound. Alternatively, a separate remnant population could (or used to) exist for *H. portusjacksoni* in this region. However, the northern coastline of Western Australia has been well explored, and the fish occurring in this region are well documented. Because no additional records of this species exist north of the Houtman Abrolhos Islands, the range of this species should not be extended north to York Sound. These records should be treated as extraneous and need further investigation.

### 4.6 Conclusion

*Heterodontus* sharks display short single oviparity, producing a single egg case per oviduct/uterus, with pairs of egg cases being deposited every 8–18 days, usually during specific breeding seasons lasting several months. Egg cases of 9 of the 10 species of *Heterodontus* are known and are disguisable from other genera by their corkscrew shape. They are separable into three morphotypes: the “wide keels lacking tendrils” group, the “narrow keels with tendrils” group, and the “wide keels with tendrils” group. Egg cases can be further distinguished to species level based on their size, the number of keel rotations, and presence of a posterior border. The collection of egg case specimens of the lesser‐known species (*H. marshallae*, *H. omanensis*, and *H. zebra*) and unknown *H. ramalheira* and their curation into biological research collections would be highly beneficial for further taxonomic and morphological studies. Observations and images of egg cases in situ are beneficial to improve the understanding of reproductive biology (laying behavior, seasonality), habitat use, and the ecological role of these sharks. Sharks kept in aquariums are also important for learning about the reproductive biology of these species and reveal information such as breeding behavior, seasonality, and incubation time.

## AUTHOR CONTRIBUTIONS

Helen L. O'Neill and William T. White conceived the study, captured and curated the data, performed the analysis, and prepared the manuscript. Kotaro Tokunaga loaned *H. zebra*, *H. francisci*, and *H. japonicus* egg case specimens and provided detailed information on the laying behaviors of *H. zebra*.

## FUNDING INFORMATION

Commonwealth Scientific and Industrial Research Organisation (CSIRO), National Research Collections Australia.
